# Preadolescent exposure to a sexually mature, unrelated male rat reduces postadolescent social recognition memory and CA2 c-Fos labeling

**DOI:** 10.3389/fnbeh.2023.1104866

**Published:** 2023-01-26

**Authors:** Teresa Maletta, Michael Palummieri, Jeff Correa, Matthew R. Holahan

**Affiliations:** Department of Neuroscience, Carleton University, Ottawa, ON, Canada

**Keywords:** hippocampus, CA2, social memory, social stress, juvenile, development

## Abstract

**Introduction:**

Social memory involves social recognition: the ability to discriminate between two or more conspecifics when one has been previously encountered. The CA2 region of the hippocampus has been implicated in social memory, as lesions and dysfunction to this area lead to social memory impairments. A variety of psychogenic manipulations during postnatal sensitive developmental periods are associated with social memory impairments later in life.

**Methods:**

In this study, we exposed preadolescent rats to a sexually, mature unrelated male and examined whether this was associated with changes in postadolescent social memory and c-Fos labeling in the CA2 region. Male and female Long-Evans rats were exposed to a male, adult rat on postnatal days 19–21 (P19–21). Social memory was measured during the postadolescent period and defined as increased interactions towards a novel age-matched rat in contrast to a previously-encountered age-matched rat. After the test, rats were euthanized and brain tissue was then collected to quantify c-Fos labeling within the CA2 region.

**Results:**

Compared to home cage controls and controls not exposed to the adult male, male and female rats exposed to the unrelated adult during preadolescence were unable to discriminate between a novel and previously encountered conspecific during the postadolescent test showing social memory deficits. The groups that showed social recognition deficits also had significantly fewer c-Fos-positive cells within the CA2 region compared to the control groups.

**Discussion:**

These findings indicate that threatening psychogenic encounters during preadolescence can have detrimental long-term effects on social memory potentially *via* disrupted activity in the CA2 hippocampal region.

## 1. Introduction

Investigations into hippocampal ontogeny and the influence of intrinsic and extrinsic factors on its developmental trajectory and neurobehavioral outcomes have gained more attention (Birnie and Baram, 2022; Ohana et al., 2022). A rich literature has found that interfering with hippocampal function during postnatal days 19–21 (P19–21) impede optimal hippocampal development and disrupt the emergence of hippocampal-based behavioral functions such as spatial learning and memory (Holahan et al., 2019; Nadel, 2022). The influence of environmental signals on the maturation of hippocampal circuits that underlie social memory, however, remains poorly understood.

One behavioral function that the hippocampus is required for is proper processing of social memories or social recognition (Maaswinkel et al., 1996; Kogan et al., 2000). Investigations have implicated the CA2 dorsal hippocampal region as being paramount to social memory processes (Hitti and Siegelbaum, 2014; Stevenson and Caldwell, 2014; Smith et al., 2016; Meira et al., 2018; Tzakis and Holahan, 2019; Oliva et al., 2020). In rodent sociability tests, rodents are exposed to a stimulus rodent for 5 min then they are reintroduced to this rodent with a novel rodent 30 min later (Feifel et al., 2009; Lukas and de Jong, 2015). In one use of this procedure, control mice spent significantly more time with the novel mouse than the familiar one, while CA2 hippocampal-lesioned mice spent similar amounts of time investigating both mice (Stevenson and Caldwell, 2014). In another, genetically-targeted silencing of CA2 neurons impaired social memory without affecting sociability or spatial and contextual memory formation (Hitti and Siegelbaum, 2014). Finally, optogenetic excitation of vasopressin terminals in the CA2 region enhanced social memory while administration of an AVPR1b antagonist directly into the CA2 impaired the enhanced social memory (Smith et al., 2016).

Numerous studies have concluded that early life stress impairs social recognition memory in adulthood using the procedure described above (Lukas et al., 2011; Kohl et al., 2015; Rincón-Cortés and Sullivan, 2016). The lack of discrimination between the novel and familiar conspecifics due to early life manipulations has been shown to not be due to changes in sociability, exploration, or olfaction, as rats still have preference for social stimuli over an object; however, when presented with two social stimuli, they are not able to discriminate (Kohl et al., 2015; Hodges et al., 2017). Thus, there is a strong case that hippocampus-dependent social memory is impaired due to early life stress, yet the involvement of the CA2 has not been explored.

The present study manipulated the social environment during a sensitive period of hippocampal development (preadolescence; P19–P21) to assess outcomes on social recognition memory and associated changes in CA2 functionality as assessed with c-Fos labeling during postadolescence. By using the Purkinje cell protein 4 (PCP4) immunostaining, a marker prominent in the CA2, it was determined that the CA2 begins its developmental trajectory around P4–5 and matures by P21 (San Antonio et al., 2014). It is hypothesized that manipulation during this time would interfere with proper CA2 development and impair behaviors dependent on this region. One study found that rat pups exposed to alcohol from P2–10 impaired their social recognition memory during adulthood (P60, P100) (Kelly and Tran, 1997). This study showed that early manipulations can impede social memory in adulthood.

The aim of the current study was to determine if impairments in social recognition memory during postadolescence would result from preadolescent exposure to an adult male. Male adult rats are considered predators to preadolescent rats and thus can be viewed as a stressor (Wiedenmayer and Barr, 2001b). Exposure to the adult male occurred during a sensitive period for hippocampal development (Holahan et al., 2019) from P19–P21. Rats were then tested on social memory in adulthood (P60 or P61) to determine if there were any social memory deficits. c-Fos positive cells were quantified within the CA2 hippocampal region to determine whether social memory impairments might be associated with reduced CA2 c-Fos labeling. It was hypothesized that preadolescent exposure to the adult male would reduce social recognition in postadolescence which would be associated with reduced CA2 activity measured *via* c-Fos positive cell density.

## 2. Materials and methods

### 2.1. Subjects

Timed pregnant (13-day gestation) female Long Evans rats (*N* = 5) were delivered from Charles River Laboratory, Quebec. They were housed in temperature (24°C) and light-controlled (8:00–20:00) Plexiglas cages with food and water *ad libitum*. The day the pups were born was marked as post-natal day zero (P0). At P18, pups (*N* = 48) were weaned, sexed, and randomly assigned into conditions. Rats from the same litter were placed in one of the following conditions: Female Stress (*n* = 8), Male Stress (*n* = 8), Female Handle (*n* = 8), Male Handle (*n* = 8), Female No Contact (*n* = 8), and Male No Contact (*n* = 8). At most, 2 rats from the same litter were assigned to the same condition. Pups within conditions were housed in pairs, given an enrichment tube, and *ad libitum* access to food and water for the remainder of the experiment. In several studies examining the developmental emergence of hippocampal function [see Holahan et al. (2019) for list of references], we have consistently weaned rats at P18 with no adverse outcomes. It is also important to note that all rats in this study were weaned at P18 so if any adverse effects did occur from weaning at P18, they would occur in all groups similarly. All procedures were approved by the Carleton University Animal Care Committee.

### 2.2. Adult-exposure regimen

**FIGURE 1:**
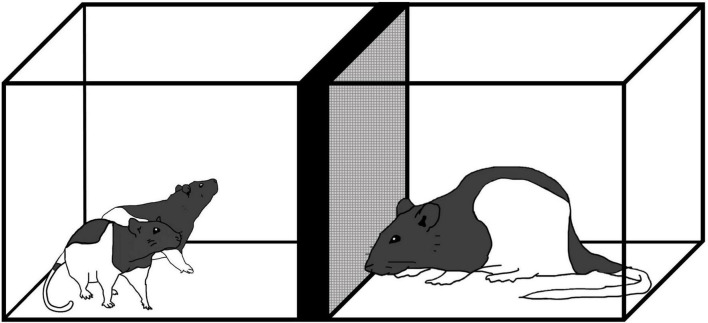
Exposure to an unfamiliar adult male rat where preadolescent rats (P19–21) were exposed to an adult male for 5 min daily from P19–P21.

From P19–21, rats in the stress groups were exposed to a sexually mature, adult male rat as described in Wiedenmayer and Barr (2001b). Use of this procedure is based on the idea that adult male rats may engage in infanticide when confronted with rat pups. In this situation, unfamiliar adult male rats engage in aggressive behavior toward rat pups naturally so there is no need to prep the adults to be aggressive. In rat pups, stimuli associated with male adult rats induces increased release of corticosterone and reduces neurogenesis (Tanapat et al., 1998). It is important that both male sex and unfamiliarity cues are required to elicit defensive behaviors in preadolescent rats (Takahashi, 1994). Examining the developmental time course of defensive behaviors in preadolescent rats exposed to an unfamiliar male shows that immobility (i.e., freezing) occurs from P14 up until P21 (Wiedenmayer and Barr, 1998, 2001a,b). The exposure procedure was carried out in a separate room in a Plexiglas cage (25 cm × 20 cm × 18 cm) with a wire mesh barrier (20 cm × 18 cm) that split the arena in half (Figure 1). Each day, pups and their respective cage mate were placed in one section of the empty arena for 10 min to habituate; afterward an unfamiliar, adult male rat (Charles River, QB) was placed in the other half of the cage. For 5 min, pups were exposed to the adult male where they could interact between the barrier. Controls were either handled for 5 min (Handle) or were left alone (No Contact) from P19–21.

### 2.3. Social recognition test

**FIGURE 2:**
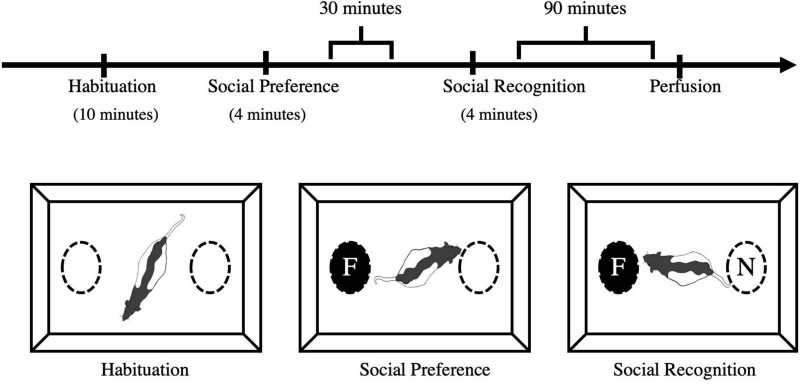
Social preference and recognition schedule and apparatus. Experimental animals were initially habituated to the apparatus for 10 min. The acquisition phase was 4 min whereby test rats were exposed to a novel conspecific in a wire mesh cage. Thirty (30) minutes after the acquisition phase, test rats were presented with the familiar conspecific and a novel conspecific and tested for their social recognition memory. Tissue was collected *via* transcardial perfusion 90 min after social recognition. F, familiar conspecific; N, novel conspecific.

At P60 or P61, each rat was subjected to the social recognition test. The apparatus was an open field (45 cm × 30 cm × 40 cm) with two cylindrical wire mesh cages (11.5 cm × 13.9 cm × 11.0 cm) placed in separate zones of the arena (Figure 2). The apparatus was situated in a standard rodent testing room with lighting and noise levels and temperature guidelines as recommended by CCAC [see Facilities.pdf (ccac.ca) section 12]. In reference to this, light intensity was 325 lux 1 meter above floor level which was also the intensity of light at the level of the apparatus. The procedure followed was adapted from Feifel et al. (2009). Male and female test rats from each condition (stress, handled, no contact) were individually placed in the apparatus to habituate for 10 min. Test rats were returned to their homecage for 30 min during which time, the apparatus was cleaned with a 70% alcohol solution. Once the alcohol had evaporated, a same-sex, age-matched, naive conspecific was placed in one of the wire mesh cages. The test rat was returned to the apparatus for 4 min. During this time (acquisition), interactions with the conspecific, and the empty wire mesh cage were collected. After this exposure, test rats and the conspecific were returned to their homecages for 30 min. The apparatus was cleaned and prepared for the test phase. For the test phase, the conspecific used during the acquisition phase (now the “familiar” conspecific) was placed into one of the wire mesh cages and an unfamiliar, naive same-sex, age-matched (termed “novel”) conspecific was placed in the other wire mesh cage. Location of the familiar and novel conspecifics was counterbalanced to minimize the chances of individual preference for location. The test phase lasted 4 min during which time, amount of time the test rats spent interacting with the familiar and novel conspecifics was collected.

All sessions were recorded and manually scored for time spent interacting with each conspecific (3 cm from the wire mesh cage) and time spent within each half of the arena. The 10-min habituation session ensured there would be no preference for one side of the cage. The first 4-min acquisition session was used to determine any changes in social behavior (the total time and number of contacts with the familiar vs. empty wire mesh cage). The second 4-min test trial was used to determine the total time and number of contacts with the familiar and novel conspecifics for each test rat in each condition.

### 2.4. cFos immunohistochemistry

Ninety minutes post-test, rats were anesthetized under isoflurane and transcardially perfused with phosphate buffer saline (PBS). Brains were removed and fixed in 4% paraformaldehyde for 48 h. Brains were then stored in a 30% sucrose solution in 0.1M phosphate buffer (PB) at 4°C. Brains were sectioned at 60 μm with a Lecica CM1900 cryostat (Welztler, Germany). Sections were labeled for c-Fos using nickel-enhanced 3,3′-Diaminobenzidine (DAB). Briefly, sections underwent three washes for 5 min in a 0.1% Triton X solution (PBS-Tx) and placed in a 0.3% hydrogen peroxide/PBS-Tx solution. Three 5-min PBS-Tx washes were followed by a 10-min incubation in animal-free blocker (AFB, Vector Laboratories). After another PBS-Tx wash, incubation in the primary antibody (monoclonal mouse anti-c-Fos IgG; PhosphoSolutions, catalog #309-cFos; 1:5000) occurred on a rocker table set at low overnight at room temperature. The next day, the tissue went through three, 10-min washes in PBS-Tx before incubating in the secondary antibody [biotinylated goat anti-mouse IgG, (H+L), Vector Laboratories; 1:500] for 2 h on a rocker table at room temperature. Three 10-min PBS-Tx washes were followed by an incubation in ABC solution (Vector Laboratories). The tissue was washed in PBS three times for 5 min and placed in a nickel-enhanced DAB solution for approximately 6 min. The slices were washed in PBS two more times for 5 min before being mounted on slides and cover slipped.

Stereology is an unbiased method that ensures any cell has equal probability of being counted while systematically accounting for total number of cells within a 3-dimensional space (West, 2002). Due to the ability to account for the z-axis, there is no need to account for size, shape, or orientation of cells; factors that need to be considered for 2-D methods and are subjected to biases (West, 2002). Thus, due to the unbiased and systematic nature of this design, we utilized stereology for cell counting. Three sections were selected from each subject to estimate the mean density of c-Fos positive cells present in the CA2 of the dorsal hippocampus. The CA2 was traced at 2.5x magnification and using unbiased stereology under Stereo Investigator 2019 (MBF Bioscience, Williston, VT, USA) cell counts were conducted at 60x magnification. c-Fos-positive cells were also counted in the CA1 and CA3 regions for comparison.

### 2.5. Statistical analyses

All data are presented as the mean ± SEM with statistical significance set at *p* < 0.05. For all behavioral and histological data, 3 × 2 Fixed-Factor ANOVAs (Stress vs. Handle vs. No Contact × Male vs. Female) were carried out followed by Fisher’s Least Significant Difference (FLSD) *post-hoc* tests.

During the acquisition and test phases, one measure that was collected was time spent in each half of the apparatus. During acquisition, it was of interest to compare time spent in the zone with the rat compared to time spent in the zone with the empty wire cage. During the test phase, it was of interest to compare time spent in the zone with the familiar rat and time spent in the zone with the novel rat. Percent time spent in these zones was recorded and a preference index was calculated (Equation 1). Multivariate fixed-factor ANOVAs (Stress vs. Handle vs. No Contact × Male vs. Female) were carried out followed by Fisher’s Least Significant Difference (FLSD) *post-hoc* tests. Unpaired *t*-tests were used to determine if the percent time spent on the side with the rat (vs. no rat; acquisition) or the side with the familiar rat (vs. novel; test) was significantly different from chance (0.5).


$$\text{Zone preference index}=\frac{\text{time spent in rat }\left(\text{acq}\right)\text{or novel }\left(\text{test}\right)\text{ zone}}{\text{total time in both zones}}$$

**Equation 1: Calculation of zone preference index.**

Specific to the 4-min acquisition phase, an analysis was carried out to determine if there was any preference for the conspecific in the wire mesh cage compared to the empty wire mesh cage. An interaction preference index (Equation 2) was calculated for time spent interacting with the cage with the conspecific rat and time spent interacting with the empty wire mesh cage. A multivariate 3 × 2 fixed-factor ANOVA (Stress vs. Handle vs. No Contact × Male vs. Female) was used followed by Fisher’s Least Significant Difference (FLSD) *post-hoc* tests. Unpaired *t*-tests were used to determine if the interaction preference index for each group and sex significantly differed from chance (0.5).


$$\begin{array}{l} \text{Interaction preference index}=\\ \frac{\text{time spent interacting with conspecific}}{\text{total time spent interacting}} \end{array}$$

**Equation 2: Calculation of interaction preference index.**

During the 4-min test phase, the test rat was placed into the apparatus with the familiar conspecific and a novel conspecific to determine if any group had changes in social recognition memory. A discrimination index (Equation 3) was calculated for time spent interacting with the wire mesh cage with the familiar rat in it and the wire mesh cage with the novel rat in it. A multivariate 3 × 2 fixed-factor ANOVA (Stress vs. Handle vs. No Contact × Male vs. Female) was used followed by FLSD *post-hoc* tests. Unpaired *t*-tests were used to determine if the zone or the interaction discrimination indexes of groups significantly different from chance (0.5).


$$\begin{array}{l} \text{Interaction discrimination index}=\\ \frac{\text{time spent interacting with novel conspecific}}{\text{total time spent interacting}} \end{array}$$

**Equation 3: Calculation of interaction discrimination index.**

Separate, multivariate 3 × 2 fixed-factor ANOVAs (Stress vs. Handle vs. No Contact × Male vs. Female) followed by FLSD *post-hoc* test were used to determine group differences in c-Fos density in the dorsal region of the CA2, CA1 or CA3 regions. Data are presented as the mean ± SEM with statistical significance set as *p* < 0.05.

## 3. Results

### 3.1. Preadolescent exposure to a male does not impact sociability

**FIGURE 3:**
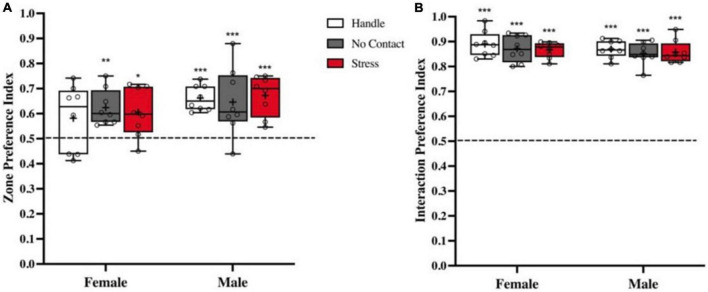
Sociability is not impacted by preadolescent exposure to unfamiliar male. Preference indexes (M ± Minimum and Maximum) comparing time spent with the conspecific vs. empty cage during acquisition. **(A)** Zone preference indexes showed that all groups in both sexes spent more time in the zone associated with the conspecific except the Female Handled group (*p < 0.05, **p < 0.01, ***p < 0.001 when compared to chance: dashed line at 0.5). **(B)** Interaction social preference indexes revealed that all conditions in both sexes spent more time interacting with the conspecific than the empty wire cage (***p < 0.001 compared to chance).

During the acquisition phase, time in the zone that contained the conspecific and time interacting with the conspecific versus the empty wire mesh cage were analyzed. There were no group differences detected for zone preference index [F(2,42) < 1.0] nor were any sex differences found [F(1,42) < 1.0] showing that all conditions spent significantly more time in the zone that contained the conspecific [Male Handle t(7) = 9.052, *p* < 0.001, Female No Contact t(7) = 4.886, *p* < 0.01, Male No Contact t(7) = 3.023, *p* < 0.05, Female Stress t(7) = 3.032, *p* < 0.05, Male Stress t(7) = 6.068, *p* < 0.001] with the exception of the Female Handle Condition t(7) = 1.74, < 1.0 (Figure 3A). To more specifically address whether preadolescent exposure affected sociability, we analyzed time spent interacting specifically with the conspecific and time spent interacting with the empty wire cage. There were no group [F(2,42) < 1.0] or sex differences [F(1,42), < 1.0] in sociability (Figure 3B). All conditions spent significantly more time interacting with the conspecific over the object [Female Handle t(7) = 21.45, *p* < 0.001; Male Handle t(7) = 30.15, *p* < 0.001, Female No Contact t(7) = 19.63, *p* < 0.001; Male No Contact t(7) = 22.46, *p* < 0.001, Female Stress t(7) = 32.78, *p* < 0.001, Male Stress t(7) = 21.95, *p* < 0.001 (Figure 3B)].

**FIGURE 4:**
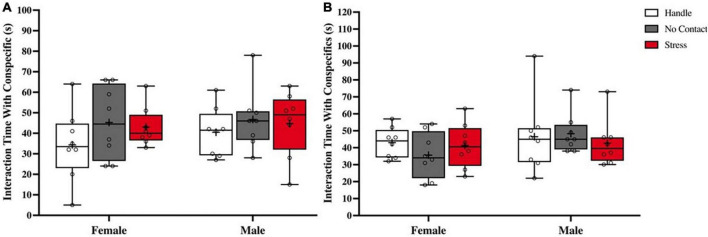
Interaction time is not altered by preadoelscent exposure to unfamiliar male. Total time interacting (M ± Minimum and Maximum) with conspecifics. **(A)** Time spent interacting with conspecific during acquisition showed no differences based on condition nor sex. **(B)** Time spent interacting with both conspecifics during social memory test showing no differences in time spent interacting with both the familiar and novel conspecifics across conditions or sex.

A third analysis during acquisition compared time spent interacting with the conspecific (Figure 4A; Data are also shown in Table 1). All groups spent equal amount of time interacting with the conspecific during the acquisition phase [F(2,45) < 1.0] (Figure 4A) with no sex differences detected [F(1,42) < 1.0] showing similar levels of interaction with the conspecific across all conditions.

**TABLE 1:** Interaction time with conspecifics during recognition test.

	Handle	No contact	Stress
	*time (s) with familiar*		
Female	13.1	12.7	22.2
Male	15.5	21.7	17.9
	*time (s) with novel*		
Female	29.9	22.9	19
Male	31.00	26.5	24.7

### 3.2. Preadolescent exposure to an adult is associated with impaired social memory

Thirty minutes after acquisition, test rats in all conditions were presented with the previous conspecific (familiar) and a novel conspecific (novel). Analysis of the time spent interacting with both the familiar and novel rats during the social memory test did not detect any group [F(2,42) < 1.0] or sex differences [F(1,42) < 1.0] (Figure 4B; Data also shown in Table 1).

**FIGURE 5:**
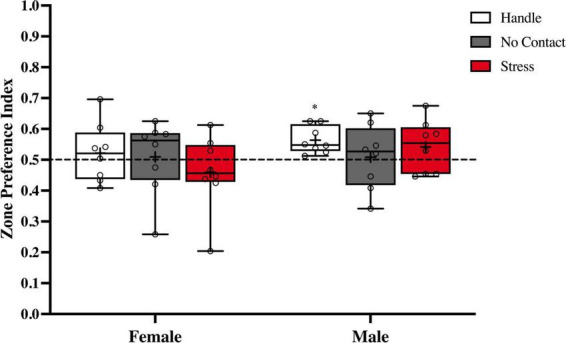
Zone preference index during the social recognition memory test. Preference indexes comparing time spent in zones with novel vs. familiar conspecifics during the social memory test (M ± Minimum and Maximum). There was no overall preference for the zone that contained the novel or familiar conspecific. *p < 0.01 Handle group versus chance.

There were no group differences in zone preference index [F(2,45) < 1.0] nor sex differences [F(1,42) < 1.0]. With the exception of the male handled group [t(7) = 4.109; *p* < 0.01] that had a marginal discrepancy from chance (*M* = 0.5635 SEM = 0.01547), there was no preference for either side of the apparatus in any other group [Female Handle t(7) = 0.6477, Female No Contact t(7) = 0.2187, Male No Contact t(7) = 0.2253, Female Stress t(7) = 0.9412, Male Stress t(7) = 1.388] (Figure 5).

**FIGURE 6:**
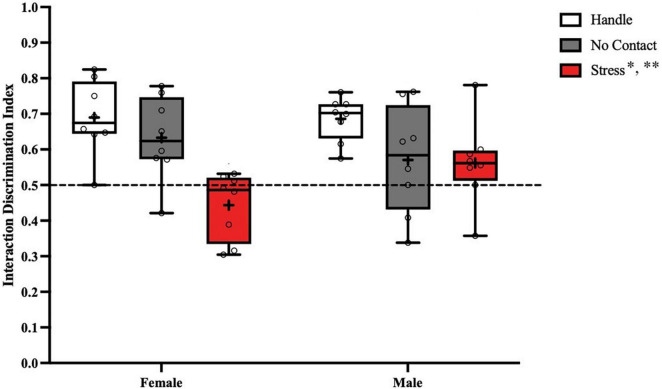
Preadolescent exposure to a male is associated with reduced social recognition memory in males and females. Both male and female groups exposed to the unfamiliar adult male during preadolescence (P19–P21) showed reduced social recognition memory during postadolescence compared to the home cage control groups (**p < 0.01) and the no contact groups (*p < 0.02).

Analysis of the discrimination index during the social recognition memory test (Figure 6) revealed a significant main effect of condition [F(2,2) = 11.08, *p* < 0.001]. No main effect was observed for sex [F(1,2) < 1.0] and no significant interaction [F(2,42) = 2.753, *p* = 0.075] was detected. FLSD *post hoc* analysis on the main effect of condition revealed that the groups exposed to the adult male during preadolescence were significantly different from the handled (*p* < 0.001) and the no contact (*p* < 0.02) groups. When followed up by one sample *t* tests, both handled groups [Female Handle: [t(7) = 5.151, *p* = 0.0013]; Male Handle: [t(7) = 8.458, *p* < 0.001], and Female No Contact [t(7) = 3.199, *p* = 0.0151] significantly differed from chance. Both groups exposed to the adult male [Female Stress: [t(7) = 1.707, *p* = 0.1315]; Male Stress [t(7) = 1.499, *p* = 0.1777], and Male No Contact [t(7) = 1.302, *p* = 0.2340] did not show any social discrimination as time spent with the novel conspecific did not differ from chance.

### 3.3. cFos labeling was reduced within the dorsal CA2 in the preadolescent adult-exposed group

**FIGURE 7:**
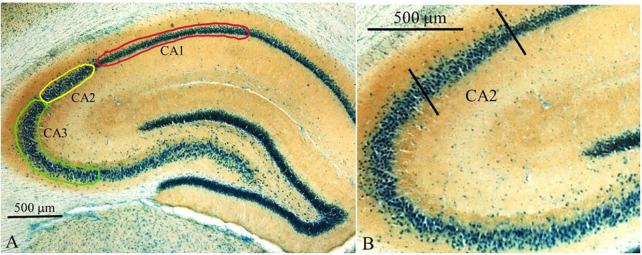
Overview of counting areas for hippocampal regions. **(A)** Lower magnification image (2.5x) of dorsal hippocampus showing the CA1, CA2, and CA3 regions. Areas where c-Fos counts were carried out are shown as outlined: green–CA3; yellow–CA2; red–CA1. **(B)** Higher power magnification (10x) delineating the CA2 region from the CA3 and CA1. Sections were stained with thionin to highlight cell bodies and to aid in the delineation of the boundary between the CA2 and CA1 subfields. Brown staining represents synaptophysin to aid with determining the boundary between the CA3 and CA2 fields.

Dorsal hippocampal subfields were delineated based on cytoarchitecture (thionin stain) and mossy fiber terminal fields (synaptophysin staining) as shown in Figure 7. Counts in each of the three main hippocampal subfields (CA1, CA2, and CA3) were carried out in areas as illustrated in Figure 7A. The CA2 region (Figure 7B) can be distinguished from the CA1 region based on cytoarchitecture and from the CA3 region based on mossy fiber terminal fields (Figure 7B and Tzakis and Holahan, 2019).

**FIGURE 8:**
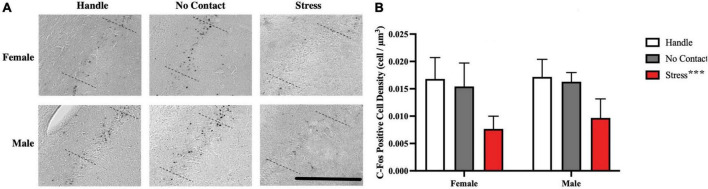
Preadolescent exposure to a male is associated with reduced c-Fos positive cell density in the CA2 region. **(A)** Sample of c-Fos positive cells observed across groups. **(B)** c-Fos positive cell density within the dorsal CA2 across conditions (M + SEM). Both male and female groups exposed to the unfamiliar adult male during preadolescence (P19–P21) showed fewer CA2 c-Fos positive neurons during postadolescence compared to the home cage control groups (***p < 0.001) and the no contact groups (***p < 0.001). Scale bar = 500 μm.

cFos-positive cells were quantified within the dorsal CA2 region of the hippocampus (Figure 8A). When comparing cell density (cell/μm^3^), it was determined that at least one group mean differed from another [F(2,2) = 30.35, *p* < 0.001]. No sex differences [F(1,2) = 1.294 *p* = 0.262] nor interaction [F(2,42) = 0.2613, *p* = 0.771] were significant. FLSD *post-hoc* analysis on the main effect of group revealed that the groups exposed to the adult male during the preadolescent period showed fewer c-Fos positive cells than the handled and no contact groups (*p* < 0.001 for both tests; see Figure 8B).

**FIGURE 9:**
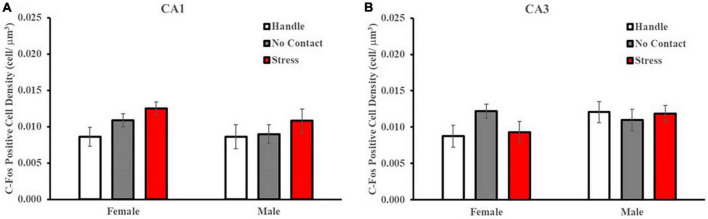
Preadolescent exposure condition does not alter c-Fos labeling in the CA1 or CA3 region of the hippocampus. c-Fos positive cell density within the dorsal CA1 and CA3 regions across conditions (M ± SEM). There were no differences detected in c-Fos labeling patterns in the CA1 **(A)** nor CA3 **(B)** regions based on group condition.

Analysis of c-Fos labeling in the CA1 (Figure 9A) and CA3 (Figure 9B) regions revealed no differences between sexes or condition. A multivariate, fixed-factor ANOVA on c-Fos positive cell density in the CA1 region revealed no main effect of sex [F(1,2) = 1.037] nor condition [F(2,42) = 2.199] nor a significant interaction [F(2,48 < 1.0). A similar analysis of CA3 c-Fos labeling revealed no main effect of sex (1,2) = 1.848] nor condition [F(2,42) < 1.0] nor a significant interaction [F(2,48) = 1.508].

## 4. Discussion

### 4.1. Sociability is not impacted by preadolescent exposure to an unfamiliar adult male

In this study, preadolescent exposure to an unfamiliar adult male did not detrimentally affect sociability as demonstrated by no change in the zone preference nor interaction preference indexes, which coincides with past literature. Others have reported that maternal separation (Franklin et al., 2011; Kohl et al., 2015; Kambali et al., 2019), social instability stress (Hodges et al., 2017), and post-weaning isolation (Naert et al., 2011) do not impact the sociability.

In contrast to these findings, maternal separation (P2–14) in female mice enhanced social preference scores in adulthood with no change observed in males (Bondar et al., 2018). Adult rats raised by prenatally-stressed mothers (P0-onward) spent significantly less time interacting with conspecifics compared to controls (de Souza et al., 2013). Peripubertal stress (P28–42) also reduced social preference in male adult rats (Márquez et al., 2013; Tzanoulinou et al., 2014). It must be noted that the impact of a stressor is dependent on both timing, duration, and form of stressor; Tzanoulinou et al. (2014) found that using the same fear-inducing stressors at different periods of development (P28–30 and P40–42) did not result in social preference differences when compared to their respective controls.

It has been found that sociability, as opposed to social memory, is not dependent on CA2 function. When Smith et al. (2016) activated Avpr1b terminals from the PVN to the CA2 in mice, there was no change in sociability, despite great enhancement in social memory. In addition, inhibiting the region does not change an individual’s sociability. Whether researchers silenced CA2 pyramidal neuron output (Hitti and Siegelbaum, 2014), lowered density of parvalbumin-expressing interneurons in CA2 *via* deletion of 22q11 (Piskorowski et al., 2016), deleted oxytocin receptors within the CA2/CA3a region (Lin et al., 2018), or inhibited of dorsal CA2 neurons through optogenetic and pharmacological means (Meira et al., 2018), sociability was not affected.

### 4.2. Social memory impairments associated with preadolescent exposure to an unfamiliar adult male

It was found that preadolescent exposure to the unfamiliar adult male significantly impaired social memory compared to the home cage control group, as both male and female adult rats were not able to distinguish between a novel and familiar conspecific. Numerous studies have documented similar findings; stressors at multiple stages of development have been linked to social memory impairments. Maternal separation (Lukas et al., 2008, 2011; Franklin et al., 2011; Reincke and Hanganu-Opatz, 2017; Kambali et al., 2019), rearing of prenatally-stressed mothers (de Souza et al., 2013), limited bedding and nesting (Kohl et al., 2015), prepubertal stress (Tzanoulinou et al., 2014), and adolescent social instability stress (Hodges et al., 2017) have all been shown to attenuate social memory. By applying the procedure developed by Wiedenmayer and Barr (2001b) we were able to target a stage of development (P19–21) that has not been previously explored with respect to later impact on social memory recognition. Unfamiliar adult male rats engage in aggressive behavior toward rat pups naturally and stimuli associated with male adult rats induces increased release of corticosterone and reduces neurogenesis (Tanapat et al., 1998) and elicits defensive behaviors in preadolescent rats (Takahashi, 1994; Wiedenmayer and Barr, 1998, 2001a,b). These reports indicate that exposure to an adult male rat male elicit a stress response which might impact the developmental trajectory of the CA2 region and impede behavioral output that depends on the intact function of this region. One such behavioral output may be social recognition memory.

Despite the preadolescent male exposure being associated with social memory deficits in both sexes when compared to the home cage control group (Handled), the Male No Contact controls did not exhibit social preference toward the novel conspecific, which was not expected. Females tend to have greater social memory (Markham and Juraska, 2007; Karlsson et al., 2015, 2016; Lawrence et al., 2015) thus perhaps the Male No Contact did not perform as well due to this sexual dymorphism. The impact on social memory recognition was specific to exposure to the male rat in females but was not specific in males such that the No Contact group, which was placed in the apparatus in the absence of the male rat, lacked intact social memory. Females are more vulnerable to trauma-related and stress-related disorders (Maeng and Shors, 2013; Li and Graham, 2017) and a body of literature demonstrate that females are more vulnerable to chronic mild stress (Dalla et al., 2005), reversed light cycles (Verma et al., 2010), prenatal stress (Grundwald et al., 2016) environmental stress (Sotiropoulos et al., 2014), and chronic unpredictable stress (McFadden et al., 2011). Thus, the threat-specific associated poor social recognition memory seen in females may likely be due to their greater vulnerability to stress. Why the males exposed to the apparatus in the absence of the unfamiliar male showed a lack in social recognition memory is not clear. There was greater variability in the social discrimination index in the Male No Contact group during the test. Wiedenmayer and Barr (2001b) found that P21 rats differentially responded to the adult male with some showing high freezing and others showing similar freezing as controls. This suggests the possibility that some male rats are more sensitive to overall changes in the environment and may show a lower threshold for a stress response. This requires further exploration.

In our work on developmental emergence of hippocampal function, we often wean our rats at P18 [e.g., (Holahan and Goheen, 2020; Tzakis et al., 2020)]. The Canadian Council on Animal Care publishes guidelines that recommend weaning to generally occur between P21–28. The guidelines state that earlier weaning is possible but pups should not be separated from the dam before P17 (Kohn and Clifford, 2002; Ito et al., 2006). In some cases, weaning prior to P17 (compared to P30) results in increased autonomic responses (e.g., heart rate) and behavioral indices of stress in males, but not females, when exposed to a novel cage but not when exposed to an unfamiliar individual (Ito et al., 2006; Kodama et al., 2008). In another study, weaning at P16 compared to P30 resulted in lower frequency of entry to and shorter duration of stay in the open arms of an elevated plus maze and in the P16-weaned rats and a tendency for P16-weaned rats to remain in the central region of an open field for shorter periods of time (Kanari et al., 2005). In mice, weaning at P21, compared to P28, was associated with more anxious and less explorative behavior in the P21 weaned mice in the open field and novel cage exposure tests (Richter et al., 2016) also see Curley et al. (2009). In the present study, rats were weaned at P18 and housed with a cagemate thereafter, which has been shown to facilitate the emergence of appropriate behavioral development (Ferdman et al., 2007; Bell et al., 2010). While we did not explore the impact of age of weaning on the immediate effects of exposure to an adult male nor the long-term outcomes, age of weaning needs to be a consideration in any studies where psychogenic manipulations are carried out. Perhaps later weaning would have a protective effect on social memory deficits that are associated with exposure to an unfamiliar adult male.

### 4.3. Reduced postadolescent CA2 c-Fos labeling associated with preadolescent male exposure

Preadolescent exposure to the adult male was associated with reduced c-Fos staining within the hippocampal CA2 region in postadolescence which was associated with the social memory impairment. No reduction in c-Fos labeling was detected in either the CA1 nor CA3 regions. This is unsurprising as social memory impairments have been related to reduction in activity of dCA2 excitatory neurons (Lin et al., 2018; Lim, 2020; Bertoni et al., 2021; Pimpinella et al., 2021; Zhou and Jia, 2021).

In contrast Young et al. (2006) were unable to demonstrate that stress could impact CA2 function. They found that acute restraint stress does not alter Avpr1b expression within the CA2; however, the form and timing of the stressor differ, making it difficult to compare. They collected brain tissue 3 h post-stress and they introduced restraint stress in adulthood rather than early in life (Young et al., 2006). The current study also has behavioral evidence to connect the reduced labeling within the CA2 region back to the expected social memory impairments. Findings from the current study show for the first time that early life stress creates conditions that contribute to social memory impairments and that reduced CA2 responses may contribute to social memory deficits. The CA2 appears to be vulnerable to stress during the preadolescent period and there are long-term impairments that persist throughout adulthood.

## Data availability statement

The raw data supporting the conclusions of this article will be made available by the authors, without undue reservation.

## Ethics statement

The animal study was reviewed and approved by Carleton University Animal Care Committee.

## Author contributions

TM designed the experiments, carried out the experiments, ran analyses, and wrote the manuscript. MP assisted with behavioral analyses and immunohistochemical staining. JC assisted with immunohistochemical analyses and statistical analyses. MH helped design the experiments and edited the manuscript. All authors contributed to the article and approved the submitted version.
